# Supplementary material for: Are carbon emissions trading and green financial instruments synergistic? -Comprehensive quantitative research based on content analysis
Source: PLoS One. 2024 Mar 7;19(3):e0298601. doi: 10.1371/journal.pone.0298601 (PMC10919601; doi:10.1371/journal.pone.0298601)
Supplement: S1 Data — (ZIP) [file pone.0298601.s001.zip › Data/地级市Z_F工作报告.zip!/║■▒▒/╧╠─■╩╨/└┤╫╘╣1⁄2╓┌║┼í╛┬φ┐╦╩2╛▌═°í┐_2010.docx]

各位代表：

现在，我代表市人民政府，向大会报告工作，请予审议，并请各位市政协委员和列席人员提出意见。

一、 2009 年工作回顾

2009 年是化危为机、砥砺奋进的一年。在省委、省政府和市委的正确领导下，全市上下深入贯彻落实科学发展观，保增长，保民生，保稳定，经济社会实现平稳较快发展。除外需不足带来出口受阻外，本届人大三次会议确定的主要目标任务圆满完成。

经济增长逆势攀升。预计生产总值 405.15 亿元、全社会固定资产投资 301.58 亿元、社会消费品零售总额 165.88 亿元、地方一般预算收入 18.14 亿元，分别增长 15.2% 、 50.1% 、 21.3% 、 27.9% 。生产总值和地方一般预算收入提前两年、全社会固定资产投资和社会消费品零售总额提前一年完成 “ 十一五 ” 目标任务。

产业结构调整加快。三次产业比重由上年的 22.8 ： 42.8 ： 34.4 调整为 21.5 ： 43.2 ： 35.3 。预计规模以上工业增加值 147.39 亿元，增长 27.3% ，提前两年完成 “ 十一五 ” 目标任务；完成第三产业增加值 143.18 亿元，增长 15.7 % 。

城乡面貌明显改观。城镇化率提高 1.5 个百分点，达到 48.1% 。 中心城区更加洁、绿、亮、美， 淦河综合治理工程荣获中国人居环境范例奖。新农村建设取得突破性进展。单位生产总值能耗和化学需氧量减排年度目标任务全面完成，二氧化硫减排提前一年完成 “ 十一五 ” 目标任务。

人民生活持续改善。预计农民人均纯收入 4873 元，增长 10.5% ，提前两年完成 “ 十一五 ” 目标任务；城镇居民人均可支配收入 11627 元，增长 9.7% 。

回顾过去一年，我们经历了风雨，见证了彩虹，为开启新的征程积累了宝贵经验，为实现新的发展奠定了坚实基 础。

（一）解放思想，提振信心，开启了 化危机、促发展的动力源泉。 深入开展学习实践科学发展观活动， 分批组团外出考察学习，再次召开建设鄂南经济强市座谈会，推动全市上下思想大解放、经济大发展。

在应急谋远中提振发展信心。 市委、市政府见微知著 ，果断决策，及时出台了促进工业、房产、金融、县域经济稳健发展的 33 条、 19 条、 16 条、 25 条措施，有效应对金融危机的冲击。 咸宁人民 登高望远，共识共为， 先后推出一批学习实践科学发展观活动鲜活经验，得到了中央、省委的充分肯定；科学确立早日建成鄂南经济强市的战略目标，绘就了 “ 五 城一区 ” 的 发展蓝图；成功举办中国 · 咸宁首届国际温泉文化旅游节， 达到了 “ 国家水平、世界影响 ” 的目标要求 ； 努力促成职教新城项目在武汉城市圈首批招标中脱颖而出，打造了咸宁跨越发展的人力资源平台；联手西门子、省联发投公司共建 “ 两型 ” 社会示范区，德中同行发展论坛走进了咸宁；积极争取上级支持，一批大产业、大工程项目纳入了全国全省投资目录，赤壁、嘉鱼、咸安挤进了武汉新港建设规划，通山列为全省脱贫奔小康试点县， 武咸城际铁路提前 21 年开工建设，武广高速铁路正式通车。 咸宁关注度日益提升，党和国家领导人、国家部委、省委省政府领导多次视察，省内外党政代表团接踵而至。 我们这座城市正在发生的深刻变化，极大地激发了全市人民的自豪感和责任感。 各县市区、市直各单位克难奋进，你追我赶 ，开创了工作新局面。 “ 两型 ” 社会综改试验、秋冬农业开发、投资、交通、国土、计生、统计、老龄等工作得到省委、省政府的充分肯 定，全省造林绿化、新农村建设、农民专业合作组织发展、农田水利基本建设、 “ 百镇千村 ” 工程、交通 “ 廉政阳光工程 ” 等现场会在咸宁召开。各类市场主体在困难面前不低头，不退缩。外商、股份制、股份合作制等规模以上企业工业增加值分别增长 18.3% 、 29.5% 、 22.4% 。民营经济贡献率提高 1.5 个百分点，达到 63% 。

在机制创新中增添发展活力。 “ 两型 ” 社会建设 综改 试验有序推进，金融、交通、旅游、教育、卫生、节能减排和生态环境建设等领域改革取得实效，改革专项总数仅次于武汉市。开发区管理体制和运行机制进一步理顺，武汉经济开发区、 “ 中国光谷 ” 咸宁 “ 园外园 ” 项目进展顺利，广东工业园、台商工业园开工建 设。 投融资机制不断创新，采用 “BOT+EPC” 方式引进中交集团投资建设咸通高速，江苏 吴江农村商业银行、美国花旗银行、黄石银 行落户咸宁， 咸安金源、开发区兴银小额贷款公司成功 组建。土地资产经营势头良好，实现土地出让金 19.05 亿元。科学理财机制不断创新，财税征管协调有序，预算管理日益完善。其他领域改革加快推进， 政府机构改革、 事业单位岗位设置管理改革全面启动，司法工作机制改革不断深化，医药卫生体制改革顺利展开，文化新闻出版机制创新得到加强，农业 “ 小三场 ” 改革有序实施，集体林权制度改革基本完成，一批老字号企业改制重组后焕发生机 ， 1 9 家党政机关所办经济实体脱钩改制稳步推进 。开放合作进一步加强。融入武汉城市圈步伐加快，厅（汉）市（县）合作成果丰硕，落实项目 263 个、资金 31.76 亿元。全市招商项目 390 个，到位资金 110.3 亿元，增长 33.9 % 。实际利用外 资 1.12 亿美元，增长 16.5% 。 咸宁与黑龙江省绥化市缔结为友好城市。

（二）扩大内需，调整结构，打造了化危机、 促发展 的强劲引擎。 以内需的扩大拉动经济增长，以结构的调整推动发展转型。

项目投资大幅增长。 四批争取新增中央投资项目 482 个，投资总额 32.5 亿元，到位中央投资 8.26 亿元，占全省 6.6% 。施工项目 1039 个，其中新开工项目 815 个，分别增长 28.4% 、 45% ，其中四个 “ 十大建设工程 ” 涉及 77 个项目，完成投资 203.2 亿元，占年计划的 108% 。红牛二期建成投产，咸宁核电、蒲圻火电二期、林浆纸一体化项目前期工作基本完成，武咸快速通道、杭瑞高速 咸宁段 、大广高速 咸宁段 、咸通高速、武赤线陶嘉一级公路、赤壁快速通道、市博物馆、职教新城、城市饮水工程、天然气管道等一批重大项目加紧推进。工业和房地产投资分别增长 24.3% 、 56% 。城镇以上固定资产投资 249.10 亿元，增长 51.4% ，增幅超历史、超全省平均水平。

市场消费持续活跃。 旅游业快速崛起。入境游客 716.74 万人次，总收入 34.03 亿元，分别增长 27.8% 、 66.3% 。城乡消费繁荣。城市、农村商品零售额达到 111.88 亿元、 54 亿元，分别增长 23.7% 、 16.4% 。商品房销售额增长 58% 。 “ 家电下乡 ” 商品销售额 1.9 亿元。市场秩序良好，物价水平稳定。

节会效应充分释放。 以节促建、以建促创、以创促变。 温泉谷、碧桂园、楚天瑶池、锦江国际、三江 温泉、通圣太乙、怡景温泉、山湖温泉等温泉宾馆、景区开业营运，投资 200 亿元、占地 54.6 平方公里的生态旅游新城初具规模。咸宁的知名度、美誉度大幅提升 。仅 去年 12 月，温泉城区十家大型宾馆接待游客人次同比增长 515% ，全市餐饮住宿营业额同比增长 120% 。

三次产业全面发展。 工业蓄势回升。规模以上企业达到 729 家，比年初净增 112 家，其中产值过亿元企业 90 家。电力能源、纺织服装、食品饮料、机械电子、森工造纸、冶金建材等六大支柱产业工业总产值 330.54 亿元，增长 29.2% ，对全市工业增长贡献率达 70% 。高新技术产业增加值 14.34 亿元，增长 18.8% 。咸宁经济开发区规模以上工业增加值 11.9 亿元，实现税收 3 亿元，分别增长 56.6% 、 50% 。县域规模以上工业增加值 125.61 亿元，增长 28.5% 。农业强基增效。粮食总产 22.5 亿斤，增长 9.5% 。特色农业板块基地新增 34 万亩，达到 321 万亩。市级以上重点龙头企业 105 家，其中省级 26 家，新增 9 家。 24 个省级示范村 “ 五个一工程 ” 建设取得突破性进展。服务业加快发展。金融机构存、贷款余额 347.19 亿元、 171.52 亿元，分别比年初增长 24.3% 、 40.3% 。担保公司 11 家，提供贷款 15.07 亿元。物流、休闲、健身、汽车、中介、信息等新兴服务业加速成长。

生态城市建设加快。 启动城市总体规划修编，编制各类规划 203 项。潜山国家森林公园二期 、南外环、青龙路、麦笠山路、 桂乡生态大道等重点工程相继建成，中心城区立面整治、拆违治超等工作有序推进， “ 四城同创 ” 深入开展，管理机制不断创新，城市功能不断完善，城市品位和容量全面提升。市区绿化覆盖率 40.4% 。嘉鱼县 、 赤壁市被评为省级园林城市。 30 家小造纸 、 小水泥 、 小炼钢企业依法关闭。

（三）改善民生，促进和谐，凝聚了化危机、 促发展 的强大合力。 着力抓好就业富民、社保利民、解困助民、稳定安民等工作，凝聚了人心和攻坚合力。

就业保障力度加大。 落实援企稳岗措施，加强就业培训指导，城镇新增就业、下岗失业人员再就业均提前完成年度目标任务。 95% 以上的返乡农民工实现再就业，应届大中专毕业生就业签约率高于上年。五项社保参保人数明显增加。城乡居民基本医疗保险制度全面实施。城乡低保、企业退休人员基本养老金、优抚对象补助提标政策全面落实。 大学生医疗保险和农村养老保险、城镇居民医保门诊统筹试点有序开展。被征地农民养老保险稳步推进。 困扰多年的陆水湖移民问题一次性解决。

社会事业明显进步。 教、科、文、卫等方面支出分别增长 33.6% 、 65.5% 、 42.2% 、 57.9% 。免收城乡义务教育学杂费，兑现义务教育学校绩效工资 ，为农村 3.78 万名义务教育贫困寄宿学生补贴生活费 2662 万元。 国家二类城市语言文字工作扎实推进。 争取国家、省重大科技专项 29 项，新增省级以上科技成果 10 项。以庆祝新中国成立六十周年为主题的群众性文化活动蓬勃开展，社会科 学、文学艺术、新闻出版、广播影视等事业日趋活跃，优秀精神产品不断涌现，外宣工作成效显著。大型民俗风情歌舞诗《梦寻咸宁》被誉为荆楚文艺奇葩。 文明创建活动深入开展，公民道德素质进一步提高。低生育水平持续稳定，人口自然增长率控制在 6‰ 以内。农产品和食品药品安全监管、安全生产和信访 维稳工作得到 加强 ，公共安全保障和应对突发事件能力不断提高，社会安定和谐。 质量振兴工作全面推进。 档案、方志等工作得到加强。人防、气象、水文、防震等工作不断改进。民族、宗教、对台、外事、侨务等工作迈出新步伐。残疾人事业有了新的进展。群团组织在社会管理服务中的作用有效发挥。

十件实事全面完成。 新 增城镇就业 3.51 万人，开发公益性岗位 2005 个，农村劳动力转移培训 2.15 万人，其中免费开展为期 3 个月的培训 1.32 万人。廉租房租赁补贴实物配租房 10731 户、发放资金 1747.4 万元，配租房源 2476 套。新增低保扩面 1.63 万人，五保 对象年供养标准提高 300 元。新 建通村公路 1604.2 公里 。建成农家书屋 226 个、乡镇综合文化站 12 个。解决农村 37.76 万人安全饮水问题；对农民购买家电按规定实行 13% 的补贴。建成 3 个县级医疗机构、 23 个乡镇卫生院和 123 个村卫生室，启动市中医院改造升级工程。启动建设 “ 九年一贯制 ” 浮山中小学。为困难群众和农民工办理法律援助 952 件。机关单位包保帮扶困难家庭 20696 户，落实帮扶资金 1226 万元；减少农村绝对贫困人口 0.6 万人、低收入贫困人口 0.53 万人。

（四）提升效能，转变作风，营造了化危机、 促发展 的良好环境。 以扎实开展 “ 作风建设年 ” 、 “ 能力建设年 ” 活动为契机，努力营造良好的政务环境。

依法行政得到加强。 自觉接受市人大的法律监督、工作监督和市政协的民主监督，认真听取各民主党派、工商联和无党派人士的意见，自觉接受新闻舆论和社会公众的监督。办理市人大代表议案和建议 90 件、市政协委员提案 120 件，办结率和见面率均达 100% 。坚持依法治市，抓好普法教育，加强行政复议和行政应诉工作，行政执法行为不断规范，综合执法试点有序推进。完善民主决策机制，重大事项集体决策、专家咨询、社会公示和听证、合法性审查等制度得到落实。推进民主管理、政务公开，电子政务建设 不断加强。认真做好国防、 “ 双拥 ” 和民兵预备役工作，军政军民关系进一步密切。

工作效能得到提升。 开展一站式集中审批、网上审批工作，方便了企业和群众。围绕确责、履责、问责，强化公务员能力席位意识，完善工作责任管理制度和绩效评估机制，强化督促检查和效能监察，加大行政问责力度，政府执行力不断提升。

作风建设得到改进。 深入开展政风行风评议活动，完善包联挂点和公开承诺服务制度。建立服务企业直通车制度，开展 “ 行风热线进开发区 ” 活动。加强经济运行分析调度，千方百计为企业排忧解难。推进节约型机关建设，行政成本明显降低。加强政府系统党风廉政建设， 行政监察和审计监督得到加强，源头治腐力度加大。

回顾过去一年，

我们深切体会到：解放思想重在解放自己，创新机制重在创造活力，应对危机重在化危为机，促进和谐重在促进民生，政府建设重在政德建设。全市人民在化危机、促发展中，表现出了不守旧、不怕难、不畏强、不服输、不气馁的良好风貌！ 今天的 咸宁，凸显了赶超快上的后发优势和美好前景！

回顾过去一年，我们清醒认识到，我市经济社会发展依然面临较大压力。金融危机的不利影响还存在，经济运行中的体制性机制性结构性矛盾还较大，民生保障压力还不小，影响社会稳定的因素还较多，公务员队伍作风建设、能力建设的任务还很重，等等。我们将进一步增强 紧迫感和使命感，采取有效措施，认真 加以解决。

各位代表！过去一年，我市经济社会发展的成绩，是在国内外经济形势严峻复杂的情况下取得的，来之不易，值得珍视。这些成绩的取得，得益于中央、省扩大内需政策的实施， 得益于市委的正确领导，得益于市人大、市政协的监督支持，得益于全市人民的团结拼搏，得益于社会各界的通力合作。在此，我谨代表市人民政府，向全市人民，向离退休老同志，向各民主党派、工商联和无党派人士，向各人民团体和企业界人士，向驻咸部队、武警官兵和政法干警，向关心支持咸宁改革发展的各界朋友，致以最崇高的敬意和最衷心的感谢！

二、 2010 年工作总体安排

今年是企稳向好成果的拓展之年，是 “ 十一五 ” 规划的收尾之年，是谋划 “ 十二五 ” 发展的基础之年。做好今年的工作，意义重大，影响深远。今年政府工作的总体要求是： 认真贯彻党的十七大、十七届三中四中全会和中央、全省经济工作会议及市委三届八次全会精神，坚持以科学发展观为指导，以建设鄂南经济强市为主线，以保持经济平稳较快发展为首要任务，以加快转变发展方式为主攻方向，牢牢把握武汉城市圈 “ 两型 ” 社会建设和长江经济带新一轮开放开发的机遇，继续解放思想，坚持务实创新，全力实施 “5610 计划 ” ，加快提高经济总量和增长质效，努力推动经济社会更好更快发展，全面超额完成 “ 十一五 ” 目标任务，为 “ 十二五 ” 发展奠定良好基础。 全市经济社会发展主要预期目标是：生产总值增长 15% 以上，规模以上工业增加值增长 24% ，单位生产总值能耗下降 4.4% ，化学需氧量和二氧化硫排放量分别下降 1.5% 、 0.5% ，全社会固定资产投资增长 35% 以上，社会消费品零售总额增长 22% ，实际利用外商直接投资增长 18% ，外贸出口增长 12% ，地方一般预算收入增长 18% 以上，城镇居民人均可支配收入增长 11% ，农民人均纯收入增长 11% ，居民消费价格涨幅控制在 3% 左右，城镇登记失业率控制在 4.6% 以内，人口自然增长率控制在 6‰ 以内。上述指标是一个预期目标，我们将以更高的目标、更实的作风、更硬的措施，力争更好的结果。

上述总体要求和预期目标的确定，主要基于以下因素：

（一）对当前经济形势的清醒判断。 新的一年，机遇十分难得。宏观经济形势总体向好，中央政策在保持连续性、稳定性的同时增强针对性、灵活性，国家大力促进中部崛起，全省加快实施 “ 两圈一带 ” 战略，沿海产业转移趋势不可逆转，特别是武广高速铁路的投入营运，成就了咸宁开放合作的新优势。新的一年，考验依然严峻。世界经济运行存在诸多变数，不排除金融风险再次冲击实体经济的可能，不排除再度出现局部金融震荡的可能。国内经济企稳回升的基础并不稳固，宏观调控形势极其复杂。我市经济运行中的困难仍然不少，特别是保增长、调结构的双重压力仍然存在。这就要求我们审时度势、攻坚克难，巩固企稳向好成果，拓展良好发展态势。

（二）对全面推进科学发展的矢志追求。 新的一年，竞争愈趋激烈。沿海发达地区经济加速转型，中西部省区及周边地市纷纷采取有力措施比环境、争客商、引项目、求发展，形成了赶超挤压的逼人态势。百舸争流，慢进亦退。咸宁位居武汉、长沙、南昌三大省会城市经济区的地理中心，处在武汉城市圈、长株潭城市群两大 “ 两型 ” 社会建设改革试验区的结合带，肩负着先行先试、争当科学发展排头兵的历史责任。这就要求我们坚持科学发展一步不错、好中求快一步不让、苦干巧干一步不虚，撑杆起跳，抢占改革发展的制高点，努力开创速度、结构、质量、效益多赢局面。

（三）对早日建成鄂南经济强市战略目标的全面对接。 新的一年，任务更加艰巨。建设鄂南经济强市第一次座谈会以来，特别是去年以来，虽然面对的困难和挑战前所未有，但我市经济社会发展仍然取得了超越预期的成绩，处在新的历史起点上。咸宁战略地位日益提升，项目建设取得重大突破，交通条件、基础设施、资源环境、产业体系不断优化，综合实力不断增强。克服困难，发扬成绩，保障 “ 十一五 ” 任务全面完成、 “ 十二五 ” 规划良好开局、鄂南经济强市早日建成，是做好今年工作的必然选择。这就要求我们继往开来、扬长补短，实现应急与谋远、打基础与建强市、保增长与调结构的有机统一，在提升发展质效的同时保持 15% 以上的增长速度。

（四）对上级党委政府和全市人民厚望的积极承载。 新的一年，使命尤为光荣。中央领导指示我们 “ 努力创造符合科学发展观要求，经得起实践、人民和历史检验的政绩 ” 。省委、省政府要求我们 “ 坚定不移地建设鄂南经济强市 ” ， “ 把咸宁建成湖北的 ‘ 岳阳 ’ ，为全省改革发展作出更大贡献 ” 。广大人民群众对我们充满期盼，希望全市经济更好更快地发展，从中得到更多更大的实惠。领导的重托、群众的呼声就是我们前进的方向。这就要求我们不骄不躁、再鼓干劲，把握好工作的方向、力度、节奏、重点，增添赶超优势，加快赶超步伐，力争发展速效高于全国、全省和武汉城市圈平均水平。

三、 2010 年工作重点

围绕今年的目标任务，我们将着力抓好以下七个方面的工作：

（一）进一步扩大内需，推动经济总量持续增长。 用足用好国家、省各项扩大内需政策，培育增长优势，做大经济总量，确保全市生产总值达到 500 亿元。

加大项目投资力度。 坚持项目立市，通过向上争取政府投资、向外引进产业投资、向内激活民间投资，力争全社会固定资产投资突破 400 亿元，工业投资和技改投资增幅均达到 30% 。注重研究国家、省投资政策和 “ 两圈一带 ” 战略规划，结合编制 “ 十二五 ” 规划，充实完善项目库，做好项目申报、争取和实施工作，保证重大项目建设有序接替。抓好已开工项目尤其是中央新增投资项目的续建和收尾，确保尽早见效。完善项目推介制度，做好对外引进项目工作。实行更加开放的投资准入政策，发挥市场机制在投融资中的作用，引导更多的社会资本进入基础设施、基础产业、高新技术、金融服务、教文体卫和公共服务领域。完善项目建设责任制度和绿色通道制度，健全领导 “ 挂企业、包项目、促发展 ” 工作制度，提高项目建设质效。

着力扩大消费需求。 发挥消费拉动经济增长的原动力作用，全面落实鼓励消费的政策措施，力争社会消费品零售总额突破 200 亿元。增强居民特别是低收入群体的消费能力，改善消费预期。深入实施 “ 万村千乡 ” 、 “ 双百 ” 、 “ 农超对接 ” 、 “ 新网 ” 工程，全面落实家电、农机、汽车摩托车 “ 三下乡 ” 和汽车、家电 “ 以旧换新 ” 等消费政策，培育农村消费热点。进一步拓宽消费领域，推进网点进社区、服务进家庭，稳步发展大宗消费，促进城市耐用品消费升级换代，大力发展商业、物业、家政、健身等服务性消费，规范发展会计、律师、公证、监理、资产评估等中介服务。优化消费环境，加强物价监测预警，建设绿色市场。

强化财政金融服务。 发挥预算资金的导向作用，优化财政支出结构，加大对民生事业、重点领域改革、重点项目建设、结构调整升级的保障力度。发挥财税政策的杠杆作用，依法加强税收征管、非税收入清理和政府投资项目评审。完善政银企合作机制和信贷政策、财政政策、产业政策协调机制，力争信贷投资规模增长 18% 以上。支持对民生工程、中小企业、 “ 三农 ” 、基础建设、节能减排、科技创新等方面的信贷服务，促进中小企业和农村融资难等问题有效缓解，保证重点项目贷款需要。落实信贷投放奖励措施，鼓励金融机构创新服务产品、优化信贷结构、提高信贷质效。加强金融监管，优化金融生态环境。

（二）进一步调整结构，促进产业优化升级。 按照传统产业高新化、高新产业规模化、优势产业特色化、特色产业现代化的思路，调整产业结构，转变发展方式。

推动工业提速增效。 坚持工业强市，按照产业抓集群、企业建龙头、产品创名牌的思路，推动各方力量向工业聚焦、各种要素向工业聚集、各项服务向工业聚合。积极实施 “ 六大支柱产业升级计划 ” ：支持和推动以咸宁核电、蒲圻火电、凯迪生物质发电等为主的电力能源产 业，以天化、精华、洁丽雅、元田等为 主的纺织服装产业，以红牛、黄鹤楼酒业、今麦郎（统一）饮品、元阳食品等为主的食品饮料产业，以合加环保设 备、三环方向机、台湾光宝、中震仪器、能一郎电池、瀛通电子、玉龙机械、吉通电瓶 等为主的机械电子产业，以林浆纸一体 化、森工科技产业园、博友竹业、金成家具、比 伦生活用纸等为主的森工造纸产业，以华新和葛洲坝水泥、华夏建龙、方圆船舶、钒矿产业、涂附磨具等为主的冶金建材产业等六大支柱产业投资扩产，技改扩 能，管理扩效，集约经营，集群发展， 确保总产值占全市工业比重 80% 以上。大力推广高新技术和先进适用技术，重点改造一批销售收入过亿元、利税过千万元的骨干企业。鼓励企业兼并重组、强强联合，打造一批龙头型、基地型、品牌型的 “ 工业代表队 ” 。继续实施中小企业成长工程，新增规模以上企业 80 家，规模以上工业增加值 180 亿元。加强重点企业运行监测分析，搞好煤电油运、重要原材料的供需衔接。支持优势产品争创品牌，新增省级以上名牌产品 12 个以上，提高 “ 咸宁制造 ” 的覆盖面和效益比。

提高农业产业化经营水平。 坚持农业稳市，大力发展特色农业、规模农业、品牌农业，加强农业综合生产能力建设，确保粮食产量稳定在 22 亿斤以上，农业总产值增长 4.5% 。完善促进农业产业化经营政策措施，按照 “ 一县一业 ” 的思路，大力实施 “ 六大特色农业品种发展计划 ” ：支持和推动咸安鸡业、嘉鱼蔬菜、赤壁猕猴桃、通山水果、崇阳鸭业、通城油茶加快发展，加快提升种养业开发经营水平，力争特色板块基地发展到 330 万亩，以 “ 特 ” 入市、以 “ 特 ” 扬名、以 “ 特 ” 取胜。大力推动菜篮子产品集约化、设施化、规模化、标准化生产。推进 “ 在山上再造一个咸宁 ” ，抓好秋冬农业开发，改造低丘岗地 10 万亩、低产林 8.5 万亩。大力发展农产品加工、销售企业，力争年销售收入过亿元的龙头企业达到 23 家。健全农业社会化服务体系，提高农民组织化程度。加强农业标准化生产，搞好动植物疫病防控，确保农产品质量安全，力争新认证 “ 三品 ” 标识产品 30 个。

促进现代服务业突破性发展。 推动现代服务业与第一、二产业互动发展，力争第三产业增加值达到 176 亿元。大力实施 “ 六大主体三产培育计划 ” ：支持和推动旅游、金融、物流、房产、信息、休闲等产业科学规划，整合开发，延伸链条，加速发展。巩固扩大办节成果，按照 “ 大旅游、大产业、大协作 ” 的要求，整合旅游资源，完善旅游要素，创建旅游精品和名镇、名村。 实施 “ 温泉 +N” 发展模式， 推动 吃、住、行、游、购、娱等 相关产业协调发展。加快推进旅游新城、汀泗桥历史文化名镇、赤壁古战场二期、龙佑温泉、五龙山温泉、九宫山风景区、太阳溪漂流、隐水洞风景区、浪口温泉度假区、山湖温泉度假区、黄龙山风景区等项目的建设与完善，构建 “1 ＋ 6” 旅游区域发展格局，力争接待游客 1000 万人次，旅游总收入增长 45% 。大力发展农家乐经济，拓宽农民增收渠道，放大旅游产业效应。支持金融创新发展，加大股份制金融机构引进力度。加强担保机构和创业投资平台建设。促进保险业稳步发展，发挥保险融资和保障功能。鼓励企业上市融资，争取上市公司有 1-2 家。改造传统商贸服务业，大力推行连锁经营、特许经营和物流配送，引进培育第三方物流企业。依托不断完善的交通运输网络，大力发展现代物流业，抓好长江产业园物流园、移动物流中心、新合作鄂南仓储配送中心、昊天建材城、 亿丰农商贸城、鄂南机电五金商贸城、 天成物流园区、嘉和明园商业城、通泰综合市场等商贸物流市场建设，努力打造华中物流枢纽、产品交易中心。加强普通商品房和保障性安居工程建设，规范发展住房二级市场，发展楼宇经济，促进房地产业健康发展。培育电子配送、网上交易等新型业态，促进电信网、互联网和广电网 “ 三网融合 ” ，加快 3G 业务发展，打造数字物流。加强信息平台建设，扩大广 播、影视、报业等 传媒和电 话、邮政、互 联网 等 网络覆盖率。结合地热资源开发利用，大力发展融体育健身、文娱表演、商务会展、文化创意于一体的休闲产业。引导社会资本投资，完善基础设施建设，培育市场主体，推动休闲产业健康发展。

加大科技创新力度。 强化企业技术创新主体地位，引导企业加大技术改造升级力度，争取 15 家企业进入全省成长路线图、创新型企业、重点示范企业等科技计划第一阵营。全面推进科技投融资、科技型与创新型企业建设、农业科技创新体系建设、科技园区建设，抓好主导产业和新兴产业领域共性技术、关键技术的攻关，为产业结构调整提供技术支撑，科技进步贡献率达到 55% 。落实支持自主创新政策措施，加大科技投入，积极引进研发机构、申报科技专项，加快高新技术产业发展，确保高新技术产业增加值增长 20% 。加强知识产权保护力度。加强与高等院校、科研院所的合作，拓展产学研合作广度和深度。

（三）进一步优化布局，促进区域协调发展。 以更大的决心、更强的力度、更高的水平，优化功能布局，促进区域协调发展，加快形成新的区域经济增长极。

加快推进 “ 五城一区 ” 建设。 加快推进工业新城建设，进一步完善咸宁经济开发区管理机制，坚持大项目立园，确保投产、新建项目各 20 个，投资、产值均增长 30% ，实现税收 4 亿元。加快推进 旅游新城建设，做好温泉资源开发保护工作，延伸旅游产业链条，加快建设山水生态居住区、森林温泉养生区、郊野运动休闲区和桂乡民俗体验区等重点项目。加快推进职教新城建设， 完成启动区基础设施配套，加快主体工程建设，完成投资 3 亿元；引进 1 － 2 所市外高校或中职学校落户，推进市区中职学校实质性整合。 加快推进梓山湖生态新城建设，实施项目详规，启动水电路等基础设施、旅游项目、被征地农民还建小区建设，完成投资 10 亿元。 加快推进咸安新城建设， 启动 107 国道城区段拓宽工程，完成三元路建设，加快建设商贸物流市场。加快推进向阳湖现代农业示范区建设，完善总体规划，制定区域性控制详规，启动一期基础设施、向阳湖文化名人中心建设，引进 1 － 2 个农业科技示范项目落户。

大力促进县域经济发展。 抢抓长江经济带新一轮开放开发和武汉新港建设的机遇，坚持市县共建、县县共建、沿江突破、垂江延伸，打造临城、临江、临港、临高速铁路、临高速公路经济带。支持咸安区抓好 “ 四区一城一带 ” 和旅游、物流、纺织、森工、食品等产业建设，支持嘉 鱼县抓好以精细化工、水泥建材、钢管制造、船舶制造、农副产品深加工等产业建设，支持赤壁市抓好 “ 一区三园 ” 和纺织服装、机电、能源、建材、造纸、旅游等产业建设，支持通城县抓好 “ 两翼四区 ” 和钒业、陶瓷、电子等产业建设，支持崇阳县抓好 “ 新天府 ” 规划和食品、建材、矿产、医药卫材等产业建设，支持通山县抓好 “ 一区两园 ” 和旅游、石材产业建设。力争赤壁市进入全省县域经济综合排名前 10 名，咸安区进入前 20 名，嘉鱼县、通城县、通山县在第二方阵中进位，崇阳县进入第二方阵。

积极推进新型城镇化 。坚持大手笔、高起点、全覆盖、严控制，健全城乡规划体系。在加快中心城市建设的同时，注重发挥中小城镇在联结城乡、辐射农村、承载产业、扩大就业中的重要作用，扎实推进县市城区、重点镇和口子镇建设，打造中小城市群，提高城镇综合承载能力。加快推进户籍制度改革，使在城镇稳定就业居住的农民有序转为城镇居民。

（四）进一步夯实基础，增强可持续发展能力。 按照打基础、利长远、惠民生的要求，加大生态保护、环境治理力度，加快推进基础设施建设，努力实现可持续发展。

加强生态环境建设。 坚持生态兴市，深入开展 “ 蓝天、碧水、绿地、宁静、清洁 ” 行动，大力推进省级环保模范城市创建。全面实施淦河生态整治、潜山森林公园三期、低碳经济示范区、金桂湖未来人居工程、咸宁垃圾焚烧发电厂、县市污水处理厂建设等 “ 六大生态环境建设计划 ” 。与华彬集团携手共进，加快培育低碳产业，积极发展低碳经济。严禁新上 “ 两高一资 ” 项目，加快淘汰落后产能。突出抓好森林资源和生物多样性保护。统筹城乡林业一体化建设，加强重点水域保护开发。逐步建立生态环境建设长效机制，探索环境资源有偿使用制度和排污权有偿交易制度。强化节能减排目标责任制管理，确保节能减排 “ 十一五 ” 目标任务全面完成。

推进基础设施建设。 突出抓好中心城市建设、交通路网建设、沿江港口建设、城乡电网扩容扩建、城乡通信网扩容扩建、水利基础设施建设等 “ 六大基础设施建设计划 ” 。进一步完善城市功能，建设潜山南入口广场、东环线、西环线、武广站中大道、金桂路延伸线、自来水三期等重点城建项目。依法推进 “ 城中村 ” 和旧城片区改造，加大土地储备力度，加强城市管理经营，实现以城建城、以城养城、以城兴城。加快杭瑞高速咸宁段、武咸城际铁路、武咸快速通道、咸通高速、通界高速、沿江一级公路建设，启动嘉鱼、赤壁长江大桥项目前期工作。加大港口建设力度，推进嘉鱼、赤壁综合型专业化深水码头建设，构建以港口为轴心的区域物流网络。加快城乡电网建设，新建 2 座 220 千伏、 4 座 110 千伏、 3 座 35 千伏输变电工程。加快通信网建设，提高通信能力，重点完成对旅游景区、交通要道、边远农村的通讯覆盖。推进水利基础设施建设，争取国家和省专项资金，抓好以 32 座病险水库除险加固为重点的防洪工程、以灌区续建为重点的灌溉工程、以生态环境改善为重点的水土保持工程、以小型农田水利建设为重点的高产农田建设工程、以排水设施完善为重点的城镇治渍排涝工程。

改善农村生产生活条件。 全面落实各项强农惠农政策，完善以奖代补、先建后补、以物抵资、民办公助等方式，增加农业农村投入，推进新农村建设。继续推进 “ 五改五通 ” 工程，深入实施 “ 百镇千村 ” 工程，着力抓好 20 个重点镇、 100 个示范村建设，加快 “ 绿色家园 ” 工程推进步伐，不断改善农村人居环境。以抓好全省脱贫奔小康试点为突破口，深入开展城乡结对共建活动，推进开发式扶贫，完成整村推进 “ 三个确保 ” 任务。继续落实好贫困地区和水库移民后期扶持政策。

（五）进一步深化改革，扩大对外对内开放。 以海纳百川的胸怀、改革创新的精神，积极破解发展难题，激发科学发展的动力和活力。

加快重点领域改革。 加快实施 “ 两型 ” 社会建设综改试验六个专项规划，落实三年行动计划的各项任务。推动厅（汉）市（县）合作纵深发展，放大共建效应。引导企业建立规范的现代企业制度。稳妥推进党政机关与所办工商企业脱钩改制工作，加强国有资产监督。深入推进农村各项改革，加大农村土地流转力度，鼓励发展多种形式的适度规模经营，稳步推进 “ 小三场 ” 改革。落实资源型产品价格、环保收费改革政策。执行最严格的耕地保护和节约集约用地制度，优化土地利用结构和布局，加强土地市场建设。推进城乡一体化综合试点。深化医药卫生体制改革，健全基层医疗卫生体系，巩固新型农村合作医疗成果，确保城镇医疗保险率达到 90% 以上。深化公益性和经营性文化事业单位改革，强化公共文化服务体系建设，加强文化市场监管。

着力承接产业转移。 创新承接产业转移政策措施，全方位推进招商引资，多形式承接产业转移。抢抓武广高速铁路全线开通机遇，全面融入武汉城市圈，加快接轨珠三角、港澳粤经济圈和长株潭城市群，重点引进产业带动强、财税贡献大、科技含量高、资源消耗少、环境污染小和有利于促进消费的好项目、大项目，加大企业总部、营运中心引进力度。完善招商引资考核办法，实施入驻项目后评估制度，不断提高项 目资金到位率，确保引资到位资金增长 20% ，实际利用外资超过 1.5 亿美元。

大力促进全民创业。 建立健全创业孵化、创业培训、创业指导、创业扶持 “ 四位一体 ” 机制，激发全民创业热情。大力实施 “ 市场主体增量 ” 行动，不论轻工业重工业，不论技术层次高中低，不论规模大中小，只要符合产业政策和环保要求，都放开、放手、放活发展。鼓励有实力的非公有资本进入金融服务、公用事业、基础设施等领域，鼓励农民通过发展乡村经济致富，鼓励 农民 、下岗失业人员、待业人员就业创业。抓好 “ 回归创业 ” 工程建设，促进打工经济向创业经济转变。注重做好企业家培养工作，树立一批民营企业典型。

稳定和开拓出口市场。 研究制定促进外贸出口的政策措施，加 快转变外贸增长方式，推动出口市场多元化、集约化，努力巩固扩大对外贸易， 力争出口总额突破 1 亿美元。 进一步优 化出口商品结构，在扩大机电产品出口的同时，重点培植麻棉纺织、医用卫材、机电、建材等出口基地，重点支持三环方向机、汇美达、大昇印染、通城砂带等出口企业发展，提高出口产品的质量和附加值。加快 “ 大通关 ” 进程，争取商检、海关在咸宁设立分支机构。大力发展对外工程承包、对外投资和劳务输出。

（六）进一步改善民生，促进社会和谐。 既要注重做好锦上添花的事情，更要注重做好雪中送 炭的工作，努力提高人民群众幸福指数。

千方百计扩大就业。 贯彻落实税费减免、小额贷款和就业援助等优惠政策，加大就业指导培训力度，开发公益性就业岗位，多渠道增加就业岗位，扩大就业容量。加强就业公共服务体系建设，重点做好大中专毕业生、城镇就业困难人员、农民工就业服务工作。抓好以创业促就业工作，实施援企稳岗计划，稳定就业形势。加强人力资源市场建设，妥善处理劳动争议，维护劳动者合法权益。

完善社会保障体系。 进一步提高社会保障水平， “ 五险 ” 扩面达到 150 万人以上。启动城镇企业职工养老保险省级统筹，做好企业退休人员基本养老金调整工作。 完 善城市低保制 度，提 高农村低保比例。建立健全困难群体临时救助制度和医疗救助制度。加快推进养老服务 体系建设，支持社 会力量兴办养老机 构。启 动残疾人社会保障体系建设。

加快社会事业发展。 坚持教育优先发展。优化基础教育布局，解决好困难家庭子女上学问题，促进城乡、区域教育均衡发展。大力发展职业教育，提高高等教育质量。创新人才工作模式，实施人才培养系统工程。加强医疗卫生工作，提高医疗服务质量，抓好甲型 H1N1 流感、 艾滋病、结核病、血吸虫病等传染病 和各类职业病的防治。大力发展文化事业。加快推进大剧院、博物馆、妇女儿童中心、青少年活动中心、传媒大厦等重点工程建设。完善歌舞剧团服务地方发展的功能。加大地方文化开发和文化遗产保护力度，丰富城乡文化生活，繁荣社会科学和文学艺术，发展新闻出版和广播影视业。推动群众性体育活动蓬勃开展，提升竞技体育水平。加强城乡社区建设，完善公共服务设施。继续推进 “ 四城同创 ” ，深入开展文明创建活动。建立健全计划生育利益导向机制，综合治理出生人口性别比，切实稳定低生育水平。做好第六次人口普查工作。加强国防动员、民兵预备役工作，广泛开展军民共建活动。强化青少年思想道德建设，关心妇女儿童、老龄事业，积极发展慈善事业，加强民族、宗教、对台、外事、侨务、人防、气象、地震、档案、方志、保密等工作。认真做好 “ 十二五 ” 规划研究编制工作。

做好安全维稳工作。 进一步落实安全生产责任制，切实加强重点领域、重点行业的安全监管，防止重特大安全事故发生。高度重视消防工作。加强食品药品安全监管，适时开展食品监管综合执法试点工作。继续加强应急管理体系建设，提高应急处置能力。进一步做好信访维稳工作，立足源头预防，注重事件处理，加强舆论引导，坚持依法办事。完善信访工作综合协调机制，积极开展群众工作部试点，做好网上信访工作，继续开展领导干部 “ 四访促和谐 ” 活动。加大拆迁纠纷、医患纠纷、国企改制遗留问题解决力度。抓好基层维稳、综治组织建设，加强社会治安综合治理，严厉打击违法犯罪活动，深入推进平安咸宁建设，增强人民群众的安全感。

（七）进一步提升效能，加强政府自身建设。 始终把对人民负责、让人民满意作为第一追求，切实加强政府自身改革与建设。

转变政府职能。 稳步实施政府机构改革，整合部门职能，规范机构设置，理顺职责关系。完善公共财政制度，深化部门预算、国库集中支付和政府采购制度改革，强化财政性资金绩效评价，提高财力的统筹安排和综合平衡能力。优化行政资源配置，推进机关集中办公服务。坚持政企、政资、政事、政社分开，政府与行业协会、市场中介组织分开，充分发挥社团组织在公共管理服务中的作用。

规范行政行为。 健全完善行政执法责任制、过错追究制、评议考核制，规范行政执法行为，推进城市综合执法管理。完善行政审批流程，规范行政审批行为。完善公共决策机制和论证评估、公示、听证、合法性审查等制度。推进政务公开，发展电子政务，加强政府门户网站建设。严格控制会议和文件的数量、规格和规模。完善绩效考核评估制度和统计制度，加大效能督查监察力度，促使公务员勇于担当、积极作为。

改进政风行风。 进一步端正学风，引导公务员学用结合，提高岗位能力。进一步弘扬求真务实的作风，完善调查研究、现场办公、服务承诺、限时办结和责任追究制度。进一步弘扬雷厉风行的作风，推行 “ 工作成果倒逼法 ” ，以目标倒逼进度、时间倒逼程序、社会倒逼部门、下级倒逼上级、督查倒逼落实。 完善政风行风评议机制，加大作风教育整顿力度，着力解决好少数公务员不尽职责、不讲效率、不守纪律等问题。

推进廉政建设。 认真落实 “ 一岗双责 ” ，切实抓好廉政建设，严格执行领导干部廉洁从政的各项规定。加大对改善民生等重大决策部署贯彻落实情况的监督检查力度，严肃治理工程建设、房地产开发、土地管理和矿产资源开发等领域的突出问题。深化预算执行审计、公共工程审计和经济责任审计，加强执法监察、廉政监察，深化源头治腐工作。深入开展节约型机关建设活动，严格控制公务消费。

各位代表！为民办实事是政府的天职。今年，市政府将加大投入，全面实施 “ 十大惠民工程实施计划 ” ：（ 1 ）城镇新增就业 3.2 万人，农村劳动力转移就业新增 5 万人。（ 2 ）推进新型农村社会养老保险试点，筹措资金 3400 万元，解决 5% 农村人口的养老保险问题。（ 3 ）筹措资金 9280 万元，新开工建设廉租住房 2320 套，新增租赁补贴 6367 户。（ 4 ）筹措资金 2000 万元，推进潜山国家森林公园三期建设。（ 5 ）新建通村公路 500 公里，完成投资 1 亿元。（ 6 ）建设 6 个乡镇综合文化站、 175 个农家书屋、 30 个农民体育健身工程；兴建 160 个村级综合服务社；对农民按规定购买家电实行 13% 的补贴。（ 7 ）新建 1.5 万户清洁能源工程，再解决农村 18.42 万人饮水安全问题。（ 8 ）为农村 5 万名义务教育阶段经济困难寄宿生补助生活费；对中职农村家庭困难学生和涉农中职学生减免学费；为 1.2 万名大学生发放奖助学金。（ 9 ）提高城镇居民基本医疗保险和新型农村合作医疗的补助标准，扩大门诊统筹覆盖面。（ 10 ）筹措资金 1800 万元，开工建设咸宁市未成年人救助保护中心、儿童福利中心、城市社会福利中心。

各位代表！政府的一切权力都是党和人民赋予的。能够在这样一个充满机遇和挑战的时代，为全市 288 万人民服务，我们深感使命光荣、责任重大。我们将始终自觉坚持党的领导，自觉依照法定权限和程序行使权力、履行职责，自觉接受市人大法律监督、工作监督和市政协民主监督，自觉听取各民主党派、工商联和无党派人士意见，自觉服务人民群众，切实加强服务型政府建设，努力创造无愧于党和人民的新业绩！

各位代表！风起潮涌，自当扬帆破浪；任重道远，更须策马加鞭！让我们紧密团结在以胡锦涛同志为总书记的党中央周围，全面落实科学发展观，在市委的坚强领导下，进一步提振克难攻坚的精气神，奏响争先创优的大合唱，为推动全市经济社会更好更快发展、早日建成鄂南经济强市而努力奋斗！
